# Implementing a Geriatric Assessment-Guided Rehabilitation Care Model in Community Oncology Care: Feasibility and Impact on Patient-Reported and Performance-Based Outcomes †

**DOI:** 10.3390/cancers17193274

**Published:** 2025-10-09

**Authors:** Mackenzi Pergolotti, Kelley C. Wood, Mary Hidde, Tiffany D. Kendig, Deanna Meehan, Katie Hutzayluk, Alaina M. Newell, Jessica Bertram, Ashley Lightner, Stacye Mayo, Alina Hedaya, Smith Giri, Grant R. Williams

**Affiliations:** 1 ReVital Cancer Rehabilitation, Select Medical, Mechanicsburg, PA 17055, USA; kecwood@selectmedical.com (K.C.W.); mchidde@gmail.com (M.H.); tkendig@selectmedical.com (T.D.K.); amnewell@selectmedical.com (A.M.N.); anlightner@bswrehab.com (A.L.); smayo@selectmedical.com (S.M.);; 2 Department of Occupational Therapy, University of North Carolina, Chapel Hill, NC 27599, USA; 3 Department of Occupational Therapy, Colorado State University, Fort Collins, CO 80523, USA; 4 Medpace, Inc., Denver, CO 80202, USA; 5 Kessler Rehabilitation Center, Kessler Institute for Rehabilitation, Select Medical, Chester, NJ 07930, USA; dmeehan@kessler-rehab.com (D.M.); khutzayluk@kessler-rehab.com (K.H.); 6 Department of Physical Therapy, South College, Knoxville, TN 37909, USA; 7 Outpatient Division, Baylor Scott and White Institute for Rehabilitation, Dallas, TX 75246, USA; jbertram@bswrehab.com; 8 Institute for Cancer Outcomes and Survivorship, University of Alabama at Birmingham, Birmingham, AL 35233, USA; smithgiri@uabmc.edu (S.G.); grwilliams@uabmc.edu (G.R.W.); 9 Lewis and Faye Manderson Cancer Center, Tuscaloosa, AL 35401, USA

**Keywords:** neoplasms, geriatric assessment, rehabilitation, triage, health services evaluation, patient reported outcome measures

## Abstract

**Simple Summary:**

This study assessed the feasibility of conducting an online monthly geriatric assessment (GA) to identify frailty in adults with cancer who were about to begin a new treatment at a community oncology practice. The GA classified patients into three categories: frail, pre-frail, or robust. Patients identified as frail or pre-frail were referred to outpatient cancer rehabilitation services, including physical and occupational therapy. Feedback from participants indicated that this approach was both feasible and well received. Furthermore, those who attended rehabilitation experienced significant improvements in quality of life, mobility, fitness, and strength.

**Abstract:**

Background: Adults with cancer who are pre-frail or frail are at risk of poor outcomes. Geriatric assessment (GA) is recommended to assess and manage vulnerability and risk of frailty in older adults with cancer (≥65) and to inform referrals in supportive services, including rehabilitation. Yet, adoption of the GA in community oncology practice lags, and frailty among adults younger than 65 often goes undetected and/or unaddressed. We evaluated the feasibility of a GA-guided rehabilitation care model and assessed changes in patient-reported and performance-based outcomes after rehabilitation. Methods: Adults (≥18 years) starting systemic therapy at a community oncology practice enrolled in the study. The GA was administered online and monthly for one year. Frailty/pre-frailty was identified using a previously validated 44-item index. The oncology team was notified of frail/pre-frail patients and then made referrals to outpatient rehabilitation. Feasibility outcomes (recruitment, retention, fidelity) and participant acceptability [7 items, 0–5 Likert scale] were analyzed descriptively. Patient-reported and performance-based outcomes were examined using the paired *t*-test. Results: 48% of eligible patients enrolled (N = 141), and 83% completed at least one GA. Frailty/pre-frailty was identified in 40% of the GAs, resulting in 282 referrals to rehabilitation (99% fidelity). Acceptability scores ranged from 3.5 ± 1.7 to 4.7 ± 0.6. Participants who attended rehabilitation (52%) improved significantly in outcomes measuring health-related quality of life, mobility, aerobic capacity, and strength (all *p* < 0.05). Conclusion: Implementing a GA-guided rehabilitation care model was feasible and acceptable to patients receiving systemic treatment. Those who attended rehabilitation experienced significant improvement in patient-reported and performance-based outcomes.

## 1. Introduction

Systemic cancer treatments are associated with high levels of cancer-related burden and frailty. Frailty is characterized by an increasing vulnerability to stressors, related to the accumulation of deficits, which decreases a patient’s physiologic reserve and resilience to stressors. In cancer, systemic treatment is associated with an increase in risk of frailty, which increases one’s risk of poor tolerability of cancer treatment, of unplanned hospitalization, long-term functional disability, poor quality of life, and early mortality [1,2,3,4,5]. Therefore, detecting and addressing frailty early is critical to a patient’s care experience and long-term well-being.

Recent guidelines from the American Society of Clinical Oncology (ASCO) recommend routine use of a geriatric assessment (GA) to identify vulnerability and risk of frailty and to provide targeted supportive care interventions, including rehabilitation (physical, occupational, and speech therapy [PT/OT/ST]), to treat GA impairments among older adults (65 and older) [6]. Adoption of these guidelines has the potential to enhance patient experience and reduce the risk of poor treatment tolerability and survival outcomes. However, integration of the GA and GA-guided care pathways into community oncology practice, although possible [7], remains a challenge due to a lack of access to a geriatrician and other supportive care services in the community [8]. Instead, researchers recommend a focus on self-administered geriatric assessments, with direct referrals to needed services, yet this has not been tested in a community-based setting.

While traditionally associated with older adults, frailty and GA impairments are increasingly recognized as issues across the age spectrum [9,10,11]. Emerging evidence highlights that accelerated aging and geriatric syndromes, including frailty, are not exclusively tied to chronological age [8,9]. The functional and economic burdens associated with cancer in working-age adults underscore the need for routine screening and early interventions to address impairments that may affect treatment outcomes and quality of life [12]. Furthermore, functional components of frailty, such as falls, difficulty with daily activities, and other impairments amendable to rehabilitation, were more common among younger adults. These findings challenge traditional assumptions that frailty and GA impairments primarily affect older adults and underscore the need to expand GA use to adults younger than 65.

To address these gaps, we conducted a prospective, pragmatic, single-arm, clinical trial integrating a GA-guided rehabilitation (defined here as physical, occupational, and speech therapy) care pathway in community oncology care for both older and younger adults initiating systemic treatment. We aimed to evaluate the feasibility of a GA-guided rehabilitation care model (only) and assess the impact of cancer-specialized outpatient rehabilitation services on health-related quality of life (HRQOL) and functional outcomes. We focused on HRQOL and functional outcomes due to their association with the ability to tolerate systemic treatment [13], unplanned hospitalization [14,15], chemotherapy-induced peripheral neuropathy severity [16], and mortality [7,10], although the literature supports the benefits of rehabilitation on these outcomes [16]. To support the broader adoption of GA-guided care and inform future research, we also report implementation strategies in alignment with the Exploration, Preparation, Implementation, Sustainment (EPIS) framework [17].

## 2. Materials and Methods

### 2.1. Study Design

This is a prospective pragmatic feasibility study conducted to evaluate the feasibility, acceptability, and impacts of the GA-guided rehabilitation care model for adults with cancer initiating a new line of systemic therapy. Study design and intervention reporting were guided by the Consolidated Standards of Reporting Trials (CONSORT) extension guidelines for feasibility and pilot studies [18] and the template for the intervention description and replication (TIDieR) checklist [19]. The study was approved by the Kessler Foundation Institutional Review Board (E-1132-20) and registered as a clinical trial (NCT04852575).

### 2.2. Study Population

English-speaking adults with cancer (18 years or older) initiating a new line of systemic cancer treatment (chemotherapy, immunotherapy, or hormonal therapy) at a multi-site community oncology private practice in East Brunswick, NJ, USA (Astera Cancer Care) were eligible. Astera is a physician-owned, community oncology practice with eleven medical oncology clinics, eight radiation clinics and three breast surgery locations. The care team at Astera includes medical oncology, radiation oncology, breast surgery, advanced practice providers, pharmacists, social workers, palliative care, and research. Individuals with no valid email address, had been referred for hospice care, or had self-reported inability (technical or cognitive) to complete online questionnaires were excluded.

### 2.3. Recruitment and Enrollment

From January to 31 December 2021, eligible patients were recruited during a pre-chemotherapy education session with an oncology advanced practice provider (APP) and were provided an additional opportunity to enroll if they elected to attend a pre-chemotherapy rehabilitation evaluation. As part of the pre-chemotherapy education session, APPs were trained to educate patients on the study and, when doing so in person, provide the study flyer. In addition, a reminder was added to the visit note in the electronic medical record (EMR) to support the APP’s ability to remember to introduce the study and to record patients’ interests. Those who agreed to contact the study coordinator were identified daily by the study team using an EMR-generated report, from which the study coordinator would call eligible patients within 48 h. Informed consent was obtained verbally in accordance with the ethical standards of the institutional review board. Once consented, each participant was enrolled in a REDCap (Research Electronic Data Capture) database [20,21] designed for this study. Throughout the follow-up period, the study coordinator routinely reviewed the EMR and removed study participants who had begun hospice services or passed away.

### 2.4. Data Collection

All GA data was collected using REDCap [20]. GAs were administered automatically at the time of enrollment (time point one), and monthly for 12 months (total of thirteen time periods over 12 months). Up to two weekly reminder emails and one telephone reminder were sent. Demographic (age, sex, race, ethnicity) and clinical characteristics (cancer type, stage, treatment regimen, etc.) were extracted from the oncology EMR for all participants. We did not estimate a target sample size for this study; instead, we worked towards the goals of a one-year collection and evaluating the feasibility of the process.

### 2.5. Intervention

The GA-guided rehabilitation care model included routine screening using a patient-reported GA, which was used to detect frailty and coordinate triage processes among the rehabilitation and oncology care teams. Participants referred to rehabilitation received individualized services provided by licensed physical and occupational therapists who had completed a cancer-specialized certification program provided by the ReVital Cancer Rehabilitation (Select Medical) and could elect to attend services at home (via synchronous telemedicine) or at an outpatient, in-community (as opposed to cancer center or hospital) clinic location. Physical therapy (PT) focused on improving range of motion, strength, and endurance. The goal of the visits was to maintain or improve cardiovascular capacity, physical health, and fitness. The therapy included 4 components: (1) cardiovascular, (2) strength training, (3) balance training, and (4) flexibility training. Occupational therapy (OT) focused on improving patients’ functioning in performing activities of daily living (ADL) such as bathing, food preparation, and managing medications, all and instrumental activities of daily living (IADL) such as upper extremity function, pain interference and social and vocational participation (i.e., the ability to return to work). Speech therapy (ST) focused on speech, swallowing, and language impairments. All PT/OT/ST interventions were tailored to the individual and their needs.

*GA Tool and Administration*. Monthly, participants were emailed a link to complete the online GA via REDCap. The GA used in this study, the Cancer and Aging Resilience Evaluation (CARE) GA, has been previously validated [22] and aligns with the GA recommended by ASCO [6]. Details of the CARE GA are provided in Supplement S1. The GA measures the following domains of frailty, each having been described previously [22,23]: the ability to complete daily activities, health-related quality of life, treatment side effects, and comorbid conditions.

*Frailty identification and triage.* GAs were scored by REDCap 11.0.0 software using a previously validated 44-item deficit accumulation model [9,24,25]. The frailty index score was calculated, and then scores were categorized as robust (0–0.2), pre-frail (0.21–0.35), or frail (>0.35) [24]. The study coordinator emailed a list of newly identified participants with pre-frail/frail status to the oncology team daily for review and, if appropriate, referral to cancer-specialized outpatient rehabilitation services. Referrals were placed by the oncology team using order sets available in their EMRs.

### 2.6. Implementation Strategies

Implementation strategies were selected by the study team a priori and iterated during each phase of the trial to create an iterative learning environment, maximizing implementation. In the exploration phase, strategies were focused on engaging and obtaining buy-ins from key stakeholders in the oncology and rehabilitation practice. In the planning phase, strategies were focused on identifying barriers and facilitators to the proposed intervention, developing educational materials, quality monitoring, and feedback systems to support implementation. During the implementation phase, additional strategies were employed to audit progress and provide feedback to key members of the oncology and rehabilitation teams, to centralize technical support, and to implement rounding calls regarding patients’ needs and operational challenges. In the sustainability phase, strategies were used to grow and adapt the program to meet demand—for example, expanding rehabilitation services to additional outpatient locations, therapists, and specialties (e.g., lymphedema and speech therapy), and improving systems to efficiently relay clinical data between the oncology and rehabilitation teams to optimize patient care and experience.

Strategies and examples are described in Supplement S2 according to the phase of implementation within the Exploration, Preparation, Implementation, Sustainment (EPIS) framework [17].

### 2.7. Feasibility Outcomes

Feasibility outcomes included: recruitment rate, retention rate, and fidelity. The recruitment rate was measured as the proportion of eligible patients who enrolled during the 1-year recruitment period. Retention rate was measured in three ways: (1) the proportion of enrolled participants who completed at least one GA during the 1-year follow-up period; (2) the proportion of enrolled participants who completed the GA in the months M1, M3, M6, and M12; and (3) the average number of GAs completed by each participant who completed at least 1 GA (possible range from 1 to 13). Common benchmarks for recruitment rates in similar feasibility studies are between 50 and 75% [26] and median recruitment rate is 38% [27]. Fidelity was measured as oncology clinician compliance to rehabilitation referrals and the proportion of referred participants who attended rehabilitation (conversion).

### 2.8. Acceptability Outcomes

As part of the month 12 GA, participants completed an 8-item acceptability survey that included seven six-point Likert scale items (0- completely disagree to 5- completely agree) and the average amount of time it took them to complete the GA (in mins).

### 2.9. Patient-Reported and Performance-Based Outcomes

Patient Reported Outcomes Measurement Information System (PROMIS^®^) health-related quality of life (HRQOL) outcomes included: Global Health (10 items, scored into global physical health (GPH), global mental health (GMH)), Physical Function (PF, 4-item), and Ability to Participate in Social Roles and Activities (SRA, 4-item). Each measure was scored using the appropriate T-score [28]. PROMIS measures have been developed and validated by the National Institutes of Health (NIH) [28], shown to be reliable and useful in oncology care and rehabilitation [29,30,31], and have been associated with cancer treatment and survival outcomes [9,15]. Higher T-scores indicate more of the domain being measured and have been shown to be sensitive and specific to capture improvement or decline in HRQOL during or following cancer treatment [32]. The established within-group minimal important change (MIC) is two points on the T-score scale, any change at or above this threshold is considered a clinically significant improvement [33].

Performance-based outcomes included: strength (handgrip strength [lbs.], 5-time sit-to-stand test [repetitions performed], and 30 s sit-to-stand test [repetitions performed]) [34,35,36], aerobic capacity (6-min walk test [distance, feet], and 2-min step test [repetitions performed]) [30,32], and mobility Timed Up-and-Go test [time, seconds]) [35].

### 2.10. Statistical Analyses

Feasibility endpoints were analyzed descriptively. Continuous scores are presented as mean with standard deviation or median with interquartile range (IQR). Categorical scores are presented as frequencies. Independent *t*-tests, Chi-squared or Fisher’s exact test were used to examine differences in characteristics between participants who enrolled versus those who completed at least one GA. Paired samples *t*-test (1-sided) were used to examine improvements in patient-reported and performance-based measures from initial evaluation to discharge. One-sided *t*-tests were used to evaluate pre-post differences because the primary hypothesis was that rehabilitation would result in improvement in HRQOL and functional outcomes. As a sensitivity analysis, we also examined two-sided tests and confirmed that the direction and significance of results remained consistent. Significance was determined a priori as *p* < 0.05. All analyses were performed using IBM SPSS Statistics (Version 27).

## 3. Results

### 3.1. Feasibility

**Figure 1:**
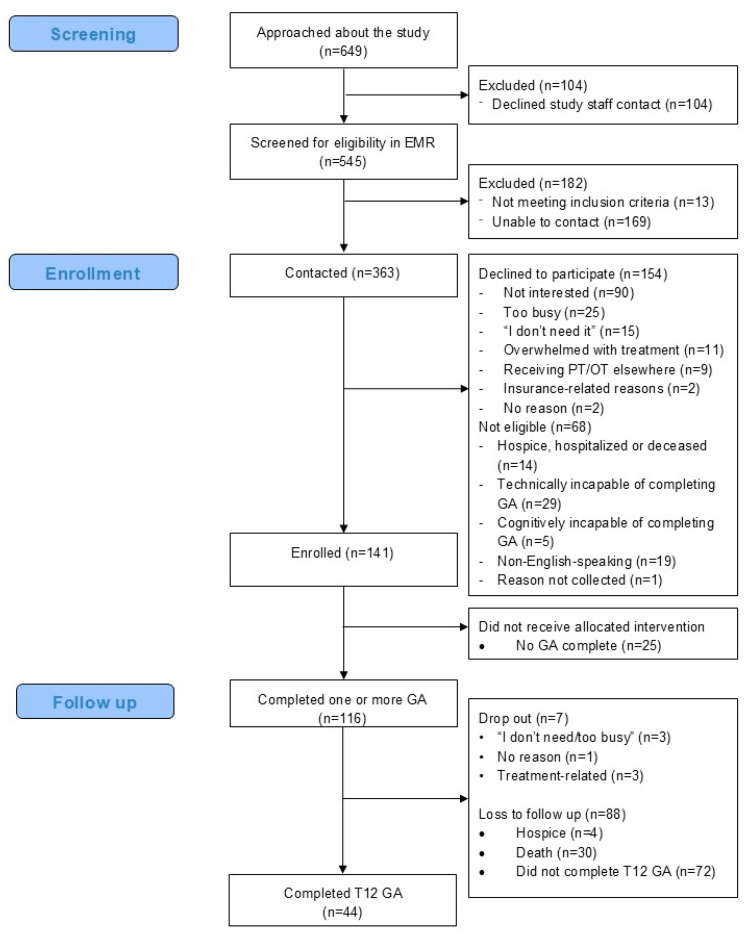
Participant screening, enrollment, and retention.

**Figure 2:**
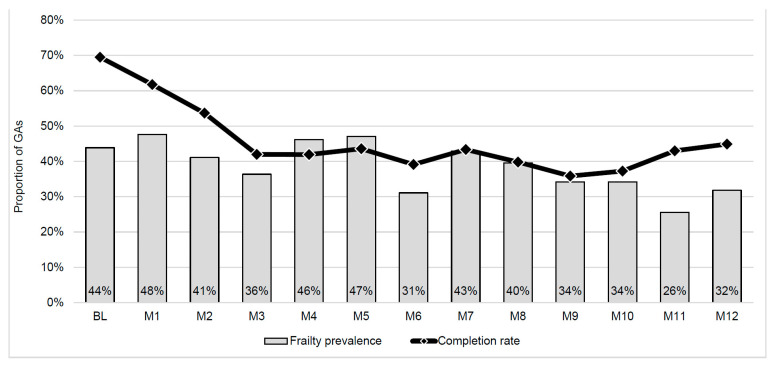
Monthly geriatric assessment completion rate and prevalence of frailty.

The recruitment rate was 48%; 141 of 245 eligible participants who were contacted enrolled in the study. Recruitment and enrollment details are provided in Figure 1 using the CONSORT extension for pilot and feasibility trials [37]. Of the enrolled patients, 51.9% were aged 64 years or younger, whereas 48.1% were aged 65 or older. Out of all the participants, 82% completed at least one GA (n = 116), and their characteristics are reported in Table 1. There were no significant differences among those who completed at least one GA and those who completed none. The monthly GA completion rate ranged from 35.9% (M9) to 69.5% (BL; Figure 2). Participants completed a median of three GAs (IQR = 1–10).

Frailty, or pre-frailty status, was identified in 40% of all completed GAs (n = 285). The monthly prevalence of frailty ranged from 26.6% (M11) to 47.6% (M1; Figure 2). Results of frailty by age and differences in reasons for frailty were published previously [23]. Twelve cases were not identified due to a technical issue in GA scoring in REDcap. Compliance with rehabilitation referrals by the oncology team was 99% (282/285 referrals). Cases not referred to rehabilitation were not eligible due to hospitalization or starting hospice. Of those referred, an additional 38 cases (13.6%) were not eligible for rehabilitation due to the following reasons: receiving home therapy (25.6%), insurance not accepted (23.1%), hospitalized (20.5%), inpatient rehabilitation (12.8%), disease progression or discontinuing treatment (7.7%), recently deceased (5%), receiving hospice care (2.6%) or moved to another state (2.6%). Conversion to rehabilitation was 51.7%. Reasons for non-conversion included not interested/do not need (74.6%), not feeling well from treatment or illness (9.1%), and receiving rehab elsewhere (9.1%). Characteristics regarding the use of rehabilitation services are reported in Table 2. No adverse events were reported due to completing GA or attending rehabilitation.

### 3.2. Participant Acceptability

Participant acceptability survey scores are reported in Table 3. The median time to complete the GA was 10 min (IQR: 5 to 10 min). Most respondents agreed the GA was simple to complete (98%), took an appropriate amount of time (95%), was helpful to monitor for treatment side effects (86%), was an added benefit to their care (81%), and that they would recommend it to peers (89%).

### 3.3. Rehabilitation Outcomes

Pre/post outcomes (any measure was included, either patient-reported or performance-based) were available for 91.3% of those who attended rehabilitation (n = 42), 31 had patient-reported outcomes, and 37 had performance-based outcomes. Statistically and clinically significant improvement, unadjusted, was observed in each patient-reported outcome (Table 4) and performance-based outcome, except for hand grip strength (Table 5). Improvement in handgrip strength was not significant (*p* = 0.183) and did not reach the MIC of 3.53 lbs. (mean change: +2.52, 11.86 lbs., CI: −8.237 to 3.195).

## 4. Discussion

Pragmatic feasibility studies are essential to test GA-guided care models recommended by the ASCO guidelines and understand how to implement them successfully. In our study, we found a non-age-restricted GA-guided rehabilitation care model was feasible and acceptable during systemic treatment in a community oncology setting with a recruitment rate of 48%, high retention rates among enrolled participants, and 99% referral compliance by the oncology team. Those who attended rehabilitation improved significantly in patient-reported and performance-based outcomes previously associated with increased chemotherapy toxicity, hospitalization, and mortality [9,14,15]. Our study adds to the growing body of literature on the integration of GA into oncology practice by testing implementation in a community oncology practice, and by focusing on integrating rehabilitation intervention. Furthermore, by not restricting participation based on age, we provide additional evidence that younger adults may also benefit from this GA-guided pathway.

GA-guided management interventions have been associated with improved chemotherapy completion [39,40], fewer grade three to five toxicities [41,42], improved patient satisfaction with patient–provider communication [43], and lower risk of short-term mortality [44]. However, connecting patients to rehabilitation continues to be a challenge [45,46,47]. Barriers have been well documented, including resource constraints, knowledge of services (when/who to refer), perceived value of services, and organizational challenges, including referring outside of a healthcare system and maintaining connection/communication with outside providers [48,49]. The GA-based EMR-integrated rehabilitation referral pathway used in this study may be a solution to previously described barriers by creating a bridge between the oncology team and cancer-specialized rehabilitation clinicians.

Building the bridge between oncology and supportive care in the real world is challenging. Our findings demonstrate the importance of the partnership between non-co located community oncology and rehabilitation services, and the potential value-add of the successful partnership between these teams in a patient’s cancer journey [50]. In addition, by reporting implementation strategies and how we used them to create an iterative learning process, we hope that the findings of this study will be helpful in informing future efforts to implement similar models of care. The partnership between the oncology and rehabilitation teams in this study was integral to success. In preparation for the study, members of the oncology and rehabilitation team worked together to design the EMR-integrated referral pathway, educational materials for patients, and reminder systems for clinicians. During the study, the rehabilitation study and oncology advanced practice provider teams communicated daily via email regarding triaged patients. The clinical rehabilitation team communicated as needed with the oncology team regarding patients’ progress or needs. The ability of the therapists and the oncology providers to communicate often, we believe, was key to helping patients feel supported and for patients to view their therapist as an extension of the oncology team.

This study also provides pragmatic evidence to understand the value of rehabilitation services during systemic treatment. Although previous randomized studies including rehabilitation and exercise interventions have shown enhanced outcomes during systemic treatment [50,51], less than 20% of patients access these services in the real world [48,49]. Due to low use, there is limited data available to truly understand the value-add of integrating these services into routine oncology care as recommended by practice guidelines and standards [52,53,54,55,56]. At baseline, participants in this study reported significant needs, one standard deviation below the norm, in terms of global physical health and physical function. This is significant due to the range of ages and types of cancer in the sample, and further demonstrates the critical need for rehabilitation during treatment. Upon discharge from rehabilitation services, patients achieved significant improvements in patient-reported outcomes of HRQOL and physical performance-based outcomes previously associated with the ability to tolerate systemic treatment [14], unplanned hospitalization [14,15], chemotherapy-induced peripheral neuropathy severity [16], and even mortality [13,14]. Interestingly, these improvements were observed despite 30% of participants being discharged from rehabilitation to hospitalization, hospice, or disease progression. Although underpowered and lacking a non-rehabilitation comparison group, when considered against the findings of previous randomized studies and longitudinal studies showing declines during treatment [57,58,59], the findings of this study provide preliminary evidence that clinically significant improvements may occur for patients who are frail/pre-frail and attending rehabilitation during systemic treatment. Interestingly, seven cases who participated in this study but were not identified as frail or pre-frail in the GA also attended rehabilitation during the 12-month follow-up period, suggesting that those considered “robust” may also have needs for and interest in receiving rehabilitation services.

### Limitations and Future Directions

While our study provides valuable insights into the feasibility and potential impact of a GA-guided rehabilitation pathway in community oncology practice, several limitations warrant consideration. Firstly, our recruitment rate of 48% may introduce selection bias, potentially influencing the generalizability of our findings. Future studies could enhance the recruitment rate by administering the GA directly from the oncology EMR or patient portal, as compared to REDcap used in this study. Secondly, although conversion to rehabilitation in this study (51.7%) was higher than in previous reports [45,47], continuing to build trusting partnerships between oncology and rehabilitation through clear communication of the value-add is needed to enhance conversion and adherence. Thirdly, this study focused solely on cancer rehabilitation needs and did not directly address other significant impairments and potential needs of patients undergoing systemic treatments, including but not limited to exercise physiology, nutrition, social support, spiritual/emotional care, financial toxicity resources, and neuropsychology, among others. We strongly recommend that future work include the expansion of the team and services beyond cancer rehabilitation as defined in this study. Finally, although improvement in rehabilitation outcomes was observed in this study, this is a small, single group study designed to evaluate feasibility. Therefore, risk for confounding and selection bias are possible in the pre/post analysis, causality cannot be determined, and generalizability may be limited. Larger adequately powered studies including a comparison or control group are needed to better understand the effects of rehabilitation for frail populations receiving cancer treatment. In the current study, the sample size was too small to account for covariates including patient age in the analysis, which has been associated with rehabilitation effects in other studies. Notably, there were insignificant differences in age between those who did (M = 62.18) and did not receive rehabilitation (M = 64.06, *p* = 0.480). Future studies should also investigate the direct effects of attending rehabilitation on additional outcomes key to healthcare quality, including unplanned hospitalization, the ability to tolerate treatment, costs, and mortality. In addition, this study informed next steps in clinical care, including creating a “prehabilitation” referral pathway for patients before chemotherapy, similar to how a prehab program would be designed before surgery, to prevent severe declines in function and potential for late- and long-lasting impairments.

## 5. Conclusions

The integration of rehabilitation services into GA-guided care models may be a practical solution to optimize patient experience and quality of life by addressing functional needs. Our study adds to the growing evidence supporting the feasibility and acceptability of integrating rehabilitation services into GA-guided clinical pathways for individuals with cancer. Future research should continue to refine implementation strategies, assess long-term outcomes, and explore the generalizability of GA-guided care models in diverse patient populations and settings. By integrating geriatric-assessment-guided rehabilitation pathways into clinical practice, oncology clinicians can optimize outcomes and improve the quality of life for individuals with cancer across the lifespan.

## Figures and Tables

**Table 1:** Participant characteristics: all enrolled versus completed at least 1 geriatric assessment (GA).

	All Enrolled (n = 141)	Completed at Least 1 GA (n = 116)	*p*-Value
Demographics			
Age	63.64 ± 10.81	64.15 ± 10.83	0.227
Sex			0.416
Male	52, 36.9%	41, 35.3%	
Female	89, 63.1%	75, 64.7%	
Race			0.871
White	88, 62.4%	77, 66.4%	
Black	14, 9.9%	12, 10.3%	
Asian	6, 4.26%	5, 4.3%	
Other	9, 6.38%	7, 6.0%	
Unknown or missing	24, 17.0%	15, 12.9%	
Ethnicity (Hispanic/Latino)	8, 5.7%	7, 7.1%	0.947
Cancer diagnosis			
Cancer type			0.179
Breast	49, 34.8%	43, 37.1%	
Head and neck	4, 2.8%	3, 2.6%	
Hematologic	25, 17.7%	20, 17.2%	
Gynecologic	9, 6.4%	7, 6.0%	
Genitourinary	7, 5.0%	7, 6.0%	
Gastrointestinal	28, 19.9%	22, 9.0%	
Lung	16, 11.3%	13, 11.2%	
Other	3, 2.1%	0, 0%	
Stage			0.646
0 to 2	44, 31.2%	38, 32.76	
3 to 4	37, 26.2%	61, 52.59%	
Unknown	23, 16.3%	14.66%	
Recurrence (at time of recruitment)	24, 17.0%	21, 18.3%	0.452
Treatment information			
First line therapy (Yes)	101, 71.6%	82, 70.7%	0.593
Number of cycles planned	11.66 ± 7.72	11.70 ± 7.88	0.892
Completed all cycles	58, 41.1%	47, 40.5%	0.748
Started new therapy during study	75, 53.2%	59, 50.9%	0.324

**Table 2:** Rehabilitation utilization characteristics, n = 46.

	n, % or Median (IQR)
Service type	
Physical therapy (PT)	39, 84.8%
Occupational therapy (OT)	1, 2.2%
Multidiscipline (PT/OT)	5, 10.9%
Speech	1, 2.2%
Insurance type	
Private	27 (57.4%)
Medicare/Medicaid/Medicare Advantage	19 (41.3%)
Rehabilitation needs ^a^	
Falls	27, 21.6%
Mobility	20, 16.0%
Pain	18, 14.4%
Weakness	16, 12.8%
Fatigue	12, 9.6%
Limited ROM	12, 9.6%
ADL limitation	10, 8.0%
Neuropathy	5, 4.0%
Other	3, 2.4%
Lymphedema	2, 1.6%
Length of stay	17.84 (5.96 to 20.71)
Visits attended	12.00 (5.50 to 25.25)
Discharge reason	
Program complete/goals met	15, 32.6%
Patient choice	11, 23.9%
Hospitalized	7, 15.2%
Surgery	3, 6.0%
Hospice	3, 6.0%
Physician discretion due to disease progression Unknown	1, 2.0% 6, 13%

^a^ Identified from International Classification of Diseases (ICD-10) codes applied by the treating therapist; up to four codes applied per case.

**Table 3:** Patient experience survey items and responses, n = 44.

Question	Average Rating (SD)	% “Agreed”
The monthly evaluation was simple to complete online	4.70 ± 0.63	97.73%
The amount of time it took me to complete the online monthly evaluation was appropriate	4.57 ± 0.97	95.45%
The online monthly evaluation was helpful to monitor for potential side effects of cancer treatment	4.16 ± 1.24	86.36%
I feel the online monthly evaluation helped me to have access to multidisciplinary care when I needed it	3.50 ± 1.72	72.72%
I would recommend the online monthly evaluation to others who are starting a new cancer treatment	4.16 ± 1.31	88.64%
The online monthly evaluation was an added benefit of receiving my care	4.07 ± 1.45	81.82%
I feel that my therapist(s) used my monthly evaluation to individualize my care *	3.79 ± 1.38	87.50%

Likert scale: (0) completely disagree, (5) completely agree. “Agreed” defined as rating ≥3 out of 5. * Answered only by those who attended rehabilitation.

**Table 4:** PROMIS^®^ Health-related quality of life outcomes of frail or pre-frail participants before and after rehabilitation.

	Specific Domains Measured	Initial Evaluation Mean, SD	Final Evaluation Mean, SD	Change Mean, SD	Change T-Score	Percent Achieving MIC
Global physical health	Overall health, ability to perform daily activities, fatigue, pain.	39.38, 7.12	43.30, 7.48	+3.92, 6.86	3.18 *	58.10%
Global mental health	Mood, ability to think, feelings of anxiety or depression, satisfaction with social activities and relationships.	45.76, 7.98	49.03, 7.17	+3.27, 7.89	2.31 *	54.80%
Physical function	Ability to do household chores, walk up/down stairs, walk at least 15 min, and run errands.	37.73, 8.35	40.79, 8.00	+3.06, 7.12	2.40 *	54.80%
Ability to participate in social roles and activities	Ability to do leisure and family activities, work, and activities with friends.	45.18, 8.11	47.49, 7.47	+2.31, 6.82	1.89 *	41.90%

n = 31. * Significant improvement in T-score from initial to final evaluation, one-sided *p* < 0.05. MIC = Minimal important change indicating clinically significant improvement (2 points) [33].

**Table 5:** Performance-based outcomes of frail or pre-frail participants before and after rehabilitation.

	n	Initial Evaluation Mean, SD	Discharge Mean, SD	Change Mean, SD	Change T-Score	MIC Value	Percent Achieving MIC
Mobility							
Timed Up-and-Go (seconds)	27	15.16, 6.23	12.81, 5.31	−2.34, 4.15	2.93 *	−1 s [35]	48.1%
Aerobic capacity							
Six-minute walk test (feet)	9	641.67, 375.77	863.44, 347.61	+221.78, 235.5	2.83 *	+65.6 feet [35]	88.9%
Two-minute step test (step repetitions)	5	57.00, 20.25	67.80, 19.72	+10.80, 5.45	4.43 *	+9.61 [38]	100%
Strength							
Handgrip strength (lbs.)	19	45.00, 12.31	47.52, 13.64	+2.52, 11.86^	0.93	+3.53 lbs [34]	47.4%
Five times sit-to-stand (seconds)	19	18.26, 5.67	15.44, 6.33	−2.82, 3.3	3.72 *	−2.3 s [35]	63.2%
30 s sit-to-stand (repetitions)	12	10.00, 4.49	12.50, 4.93	2.50, 4.81	1.80 *	+2 repetitions [36]	58.3%

* Significant improvement from initial to final evaluation, one-sided *p* < 0.05.

## Data Availability

Data and materials are available upon request from the corresponding author.

## Supplementary Materials

The following supporting information can be downloaded at: https://www.mdpi.com/article/10.3390/cancers17193274/s1, Supplement S1: Cancer and Aging Resiliency Evaluation (CARE), Supplement S2: Implementation strategies used to implement a GA-guided rehabilitation referral pathway.
