# Supplementary material for: Implementing a Geriatric Assessment-Guided Rehabilitation Care Model in Community Oncology Care: Feasibility and Impact on Patient-Reported and Performance-Based Outcomes
Source: Cancers (Basel). 2025 Oct 9;17(19):3274. doi: 10.3390/cancers17193274 (PMC12523720; doi:10.3390/cancers17193274)
Supplement: Supplementary file 1 [file cancers-17-03274-s001.zip › cancers-3828194-supplementary/Supplement S1. Cancer and Aging Resiliency Evaluation (CARE).pdf]

Cancer and Aging Resiliency Evaluation (CARE)

The CARE has been shown to be feasible to implement as a part of routine oncology care, including high patient completion rates (91%) and patient satisfaction with survey length (83%). From the CARE, a frailty index based on the principles of deficit accumulation can be derived that categorizes patients as robust, pre-frail, or frail. For this study only measures included in the frailty index have been included in the mCARE. Details of the *m*CARE below by their respective domain.

Function. The first item assesses the number of falls in the last 6 months. Next, the ability to perform Instrumental Activities of Daily Living (IADL) (6 questions) and Activities of Daily Living (ADL) (3 questions) is assessed, both from a subscale of the Multidimensional Functional Assessment Questionnaire (MFAQ): Older American Resources and Services (OARS); these questions inquire about the ability to perform IADL and ADL and responses are: without help, with some help, or completely unable to perform. Next, is a single item regarding whether health limits ability to walk one block. This physical function item along with the number of falls in the last 6 months was specifically chosen as it is part of both the CARG toxicity calculator and e-prognosis assessments (see below).

Psychological. The CARE also utilizes PROMIS assessments of anxiety (4 item), depression (4 item), and cognitive function (4 item). Both of these measures are answered on a 5-point Likert scale ranging from “Never” to “Always,” are scored on a 0-100 T-score, and have developed cross-walks to legacy instruments including PHQ-9 and GAD-7.

Cognitive function. The PROMIS 4 item assessment of Cognitive Function is used as a patient-reported screener for perceived cognitive impairment. Its validity and reliability have established to assess cognitive impairment in a sample of medical patients in the outpatient setting. PROMIS assessments have been highlighted in CARE given their validity, ease of use, thoughtful design, free of charge, and potential to adapt to computer adaptive testing (CAT) for use in future tablet based GA versions.

Nutrition is assessed via the Patient Generated – Subjective Global Assessment (PG-SGA) (http://pt-global.org). The CARE uses the shortened PG-SGA that assess weight loss, food intake, symptoms related to nutrition, and a general activities/function question. The general activities and function question closely mirrors that of the standard oncology performance status measure developed by the Eastern Cooperative Oncology Group (ECOG). The short form PG-SGA has been shown to be a practical and valid tool for detecting malnutrition and cachexia specifically in the outpatient oncology setting. The nutrition assessment is followed by the 8 item Medical Outcomes Survey (MOS) Social Support Survey that provides subscales of information and emotional support.

Comorbidity. The CARE then inquiries about the number of medications the participant takes on a daily basis and the number of comorbid conditions besides their cancer. The next section, evaluates how much the participant's physical or emotional health has interfered with their social activities then assesses overall health and hearing ability on a 5-point Likert scale. Next, is a patient-reported comorbidity scale (the Physical Health Section of the OARS) that contains a list of current illnesses and the degree to which each interferes with daily activity. This comorbidity scale has been shown to be independently predictive of overall survival in older adults with breast cancer.

Health care utilization. The following question asks whether the participant has been seen in the emergency room (ER) or hospitalized in the past year. The presence of ER or hospitalizations is included as they are part of most life expectancy calculators and can be useful as outcome measures.

Financial toxicity. A single financial toxicity screening question has also be included, from the Patient Satisfaction Questionnaire (PSQ-18) that asks whether the participant has to pay for more medical care than they can afford.
