# Supplementary material for: Implementing a Geriatric Assessment-Guided Rehabilitation Care Model in Community Oncology Care: Feasibility and Impact on Patient-Reported and Performance-Based Outcomes
Source: Cancers (Basel). 2025 Oct 9;17(19):3274. doi: 10.3390/cancers17193274 (PMC12523720; doi:10.3390/cancers17193274)
Supplement: Supplementary file 1 [file cancers-17-03274-s001.zip › cancers-3828194-supplementary/Supplement S2. Implementation strategies used to implement a GA-guided rehabilitation referral pathway.pdf]

Supplement S2. Implementation strategies used to implement a GA-guided rehabilitation referral pathway

Strategy & description	Example	Applicable EPIS Implementation Framework Stages			
		E	P	I	S
**Identify and prepare champions** *Identify and prepare individuals who dedicate themselves to supporting, marketing, and driving through an implementation, overcoming indifference or resistance that the intervention may provoke in an organization*	Oncology team members (chief medical officer, MDs and APPs) attended regular planning calls to plan the intervention and training for subsequent team members.	■			
**Conduct local consensus discussions** *Include local providers and other stakeholders in discussions that address whether the chosen problem is important and whether the clinical innovation to address it is appropriate*	Oncology president and chief medical officer identified need to improve access to rehabilitation and integrate GA and participated in ongoing planning meetings to ensure appropriateness of the intervention.	■	■		
**Build a coalition** *Recruit and cultivate relationships with partners in the implementation effort*	Key partners were identified and consulted to inform intervention design including oncology EMR experts, nurse navigators and rehabilitation scheduling staff.	■	■		
**Assess for readiness and identify barriers and facilitators** *Assess various aspects of an organization to determine its degree of readiness to implement, barriers that may impede implementation, and strengths that can be used in the implementation effort*	Frequent meetings were conducted with clinical and administrative staff to discuss facilitators and problem-solve barriers.	■	■	■	
**Mandate change** *Have leadership declare the priority of the innovation and their determination to have it implemented*	Oncology president and chief medical enforced importance with oncology admin and clinical staff during local meetings.		■		
**Develop and distribute educational materials** *Develop and format manuals, toolkits, and other supporting materials in ways that make it easier for stakeholders to learn about the innovation and for clinicians to learn how to deliver the clinical innovation, and distribute them to key stakeholders.*	Program flyers were created for patient education. Therapists completed asynchronous courses, attended synchronous presentations and were provided written materials (e.g., systemic treatment side effects and special considerations). Education materials were shared with oncology providers via the oncology EMR, with patients via email or in-clinic visits, and with therapists via a shared rehabilitation network drive.		■	■	
**Develop and organize quality monitoring systems** *Develop and organize systems and procedures that monitor clinical*	A scheduling log was created and maintained by rehabilitation admin. Study coordinator created and		■	■	■

processes and/or outcomes for the purpose of quality assurance and improvement	maintained spreadsheets to track rehabilitation use and outcomes data to facilitate regular audits and provide feedback to rehabilitation and oncology clinical teams via meetings and email distribution lists.	[redacted]
**Remind clinicians**<br>*Develop reminder systems designed to help clinicians to recall information and/or prompt them to use the clinical innovation*	A templated daily email was sent to oncology staff notifying them on new frail/pre-frail patient in need of referral and those who had been referred but were not able to be contacted or declines rehabilitation.	[redacted]
**Audit and provide feedback**<br>*Collect and summarize clinical performance data over a specified time period and give it to clinicians and administrators to monitor, evaluate, and modify provider behavior*	Case-level performance data (rehabilitation use & outcomes) was reviewed weekly with rehab clinicians. Monthly compliance reports regarding screening and referral were emailed to the oncology team.	[redacted]
Centralize technical assistance<br>*Develop and use a centralized system to deliver technical assistance focused on implementation issues*	Email distribution lists and e-mailboxes were created for technical assistance needs. Synchronous and a-synchronous training was provided to therapists conducting telehealth.	[redacted]
**Organize clinician implementation team meetings**<br>*Develop and support teams of clinicians who are implementing the innovation and give them protected time to reflect on the implementation effort, share lessons learned, and support one another's learning*	Ongoing weekly meetings Rehabilitation clinicians attended a weekly call to round on patients, problem-solve operational challenges (i.e., scheduling, telehealth), and share successes. Two oncology APP leads were identified by leadership and met monthly with the research team.	[redacted]
**Change service sites**<br>*Change the location of clinical service sites to increase access*	Rehabilitation started in four, then expanded to additional clinics to increase access to additional sites and clinical specialties (e.g., lymphedema and speech therapy). Telehealth used to expand access when in-clinic appointments were not possible due to COVID-19, patient health or transportation challenges.	[redacted]
**Create new clinical teams**<br>*Change who serves on the clinical team, adding different disciplines and different skills to make it more likely that the clinical innovation is delivered (or is more successfully delivered)*	Implementation began with a group of four therapists (physical and occupational therapy) then expanded to additional specialized therapist based on patient need (e.g., lymphedema, speech).	[redacted]
**Facilitate relay of clinical data to providers**	Clinical notes were exchanged between oncology and	[redacted]

Provide as close to real-time data as possible about key measures of process/outcomes using integrated modes/channels of communication in a way that promotes use of the targeted innovation	rehabilitation teams – including changes in patient status and progression towards treatment goals.
